# Linezolid in the era of bedaquiline-pretomanid-linezolid-based regimens: an indispensable challenge

**DOI:** 10.36416/1806-3756/e20260324

**Published:** 2026-08-13

**Authors:** Ana Paula Santos, Fernanda Carvalho de Queiroz Mello

**Affiliations:** 1.Faculdade de Ciências Médicas, Universidade do Estado do Rio de Janeiro - UERJ - Rio de Janeiro (RJ) Brasil.; 2.Instituto de Doenças do Tórax, Universidade Federal do Rio de Janeiro - UFRJ - Rio de Janeiro (RJ) Brasil.; 3.Faculdade de Medicina da Universidade Federal do Rio de Janeiro - UFRJ - Rio de Janeiro (RJ) Brasil.

Initially classified under “Group 5” of drugs for drug-resistant tuberculosis in the 2011 WHO guidelines,^1^ linezolid was rapidly reclassified as “Group A” in the 2018 update.^2^ In this revised guidance, linezolid was designated a priority agent for the treatment of drug-resistant tuberculosis, based on emerging evidence supporting its efficacy-most notably, its significant contribution to mortality reduction in patients receiving linezolid-containing regimens.^3^

Linezolid is an antimicrobial agent belonging to the oxazolidinone class, originally developed for the treatment of infections caused by resistant Gram-positive bacteria. Its mechanism of action involves the inhibition of protein synthesis at the ribosomal level in *Mycobacterium tuberculosis*, resulting in moderate bactericidal activity and pronounced sterilizing effects.^4^ Research into its application in mycobacterial infections dates to the late 1990s, when animal model studies demonstrated efficacy comparable to that of isoniazid.^5^ Subsequent *in vivo* studies corroborated its clinical efficacy in patients with drug-resistant tuberculosis.^6^ Nevertheless, prolonged use was associated with adverse events, even following dose reduction.

In this issue of Respiratory Research & Clinical Practice, Amiri et al.^7^ confirm the efficacy of linezolid for the treatment of rifampin-resistant/multidrug-resistant tuberculosis (RR/MDR-TB), whether as part of standardized or individualized regimens. Using systematic review and meta-analysis, the research team evaluated 22 studies encompassing a total of 3,014 patients across 26 comparison arms. The pooled results demonstrated that linezolid-containing regimens were associated with a favorable treatment success rate of 80.1%. However, the authors focus on the safety and tolerability of linezolid describing adverse events including myelotoxicity (33.1%), peripheral neuropathy (33.9%), psychiatric and neurological disorders (12.3%), myalgia (7.0%), optic neuropathy (7.8%), gastrointestinal events (1.9%), ototoxicity (2.9%), and rash (1.0%). Moreover, serious adverse events were rarely reported. 

According to the most recent WHO guidelines for the treatment of drug-resistant tuberculosis, linezolid is described as the most toxic drug in bedaquiline-pretomanid-linezolid (BPaL)-based regimens and carries the highest incidence of adverse events among antituberculosis agents.^4^ The spectrum of linezolid-associated adverse events is broad, ranging from mild and common manifestations-such as taste disturbance and diarrhea-to rare but severe complications, including serotonin syndrome.^4^ Peripheral neuropathy, myelotoxicity, and optic neuropathy were the most frequently reported adverse events in a Japanese retrospective cohort^8^ and was also highlighted in the study by Amiri et al.^7^ While myelotoxicity generally responded favorably to treatment discontinuation or dose reduction, peripheral neuropathy proved refractory to these interventions in 45% of cases.^8^ Evidence from a recent systematic review further establishes a clear dose- and duration-dependent relationship with these neurological manifestations.^9^ To mitigate this toxicity, therapeutic drug monitoring (TDM) has been proposed as a key strategy.^10^ However, routine TDM remains largely unavailable in the Brazilian healthcare context. 

The study by Conradie et al.,^11^ the first of the three pivotal clinical trials supporting the use of BPaL, initially employed a linezolid dose of 1,200 mg daily, based on the approved dose used for the treatment of bacterial infection. Subsequently, the reduction to 600 mg daily, and further to 300 mg daily or temporary discontinuation of linezolid, was permitted in cases of known linezolid-associated adverse events, including myelosuppression and peripheral neuropathy. By the end of the trial, 36.5% of participants completed treatment at a dose of 600 mg, 15.4% at 300 mg, and 30.7% discontinued linezolid early due to an adverse event.^11^ Subsequently, a trial evaluated BPaL regimens with linezolid at three different doses: 1,200 mg, 600 mg, or 300 mg daily. The overall risk-benefit ratio favored the group receiving the three-drug regimen with linezolid at 600 mg for 26 weeks, which demonstrated a lower incidence of adverse events and fewer dose modifications. The rate of peripheral neuropathy was 24%, while myelosuppression occurred in 2% of patients.^12^ Finally, Nyang’wa et al. demonstrated that a 24-week fully oral BPaL-plus-moxifloxacin regimen was non-inferior to the accepted standard-of-care treatment, with a more favorable safety profile.^13^

Despite the high cost of the drug, the Brazilian Ministry of Health incorporated linezolid into the standardized regimens for the treatment of RR/MDR-TB in 2021. Initially included in the fully oral, albeit lengthy, regimen in combination with bedaquiline, levofloxacin, and terizidone,^14^ and subsequently in the shortened regimen together with bedaquiline and pretomanid,^15^ linezolid was made available in its oral or parenteral formulations following patient registration in the Special TB Treatment System. Monitoring by the healthcare team for adverse events is encouraged, as is surveillance of the most frequently reported events. According to the Technical Note published in 2025, patients receiving the BPaL regimen should undergo monthly blood collection for complete blood count analysis, aimed at the early identification of any cytopenia, while clinical manifestations of peripheral and optic neuropathy should also be systematically assessed. The recommended strategy for managing adverse events varies according to their severity and the treatment window, ranging from linezolid dose reduction to treatment discontinuation and the indication of an individualized regimen. In cases of manifestations suggestive of optic neuropathy, linezolid should be promptly discontinued and an alternative regimen excluding the drug should be initiated.^15^

Another point highlighted by Amiri et al.^7^ is the need for new oxazolidinone drugs that maintain the same efficacy while presenting lower toxicity. Several new molecules showed relevant *in vitro*, *in vivo* and clinical studies, including sutezolid, tedizolid, delpazolid, eperezolid, radezolid, contezolid, posizolid, and TBI-223, as identified in the review by Chen et al.^16^ The most recent TB Alliance pipeline includes sutezolid and TBI-223 in phase II clinical trials. In the PanACEA-SUDOCU-01 study,^17^ sutezolid combined with bedaquiline, delamanid, and moxifloxacin demonstrated efficacy, increasing time to culture positivity, and without toxicities associated with the doses used. Preclinical data indicated potent antimycobacterial activity and a superior safety profile of TBI-223 compared with linezolid.^18^

Meanwhile, linezolid remains essential in short treatment regimens alongside bedaquiline and pretomanid, despite its potential toxicity. Until a novel oxazolidinone is recommended, linezolid will require careful monitoring of adverse events by the healthcare team-along with therapeutic drug monitoring when available-which are the main strategies to mitigate linezolid-associated toxicity.
